# Protective Role of 5-Lipoxigenase during *Leishmania infantum* Infection Is Associated with Th17 Subset

**DOI:** 10.1155/2014/264270

**Published:** 2014-09-21

**Authors:** Laís Amorim Sacramento, Fernando Q. Cunha, Roque Pacheco de Almeida, João Santana da Silva, Vanessa Carregaro

**Affiliations:** ^1^Department of Biochemistry and Immunology, Ribeirão Preto Medical School, University of São Paulo, Avenida Bandeirantes 3900, 14049-900 Ribeirão Preto, SP, Brazil; ^2^Department of Pharmacology, Ribeirão Preto Medical School, University of São Paulo, Ribeirão Preto, SP, Brazil

## Abstract

Visceral leishmaniasis (VL) is a chronic and fatal disease caused by *Leishmania infantum* in Brazil. Leukocyte recruitment to infected tissue is a crucial event for the control of infections such as VL. Leucotriens are lipid mediators synthesized by 5-lipoxygenase (5-LO) and they display a protective role against protozoan parasites by inducing several functions in leucocytes. We determined the role of 5-LO activity in parasite control, focusing on the inflammatory immune response against *Leishmania infantum* infection. LTB_4_ is released during *in vitro* infection. The genetic ablation of 5-LO promoted susceptibility in highly resistant mice strains, harboring more parasites into target organs. The susceptibility was related to the failure of neutrophil migration to the infectious foci. Investigating the neutrophil failure, there was a reduction of proinflammatory cytokines involved in the related Th17 axis released into the organs. Genetic ablation of 5-LO reduced the CD4^+^T cells producing IL-17, without interfering in Th1 subset. *L. infantum* failed to activate DC from 5-LO^−/−^, showing reduced surface costimulatory molecule expression and proinflammatory cytokines involved in Th17 differentiation. BLT_1_ blockage with selective antagonist interferes with DC maturation and proinflammatory cytokines release. Thus, 5-LO activation coordinates the inflammatory immune response involved in the control of VL.

## 1. Introduction

Visceral leishmaniasis (VL) is one of the most severe clinical manifestations of infection with* Leishmania* parasites and it is a major cause of human mortality and morbidity globally; VL is caused by* Leishmania donovani* and* Leishmania infantum* (World Health Organization, 2010).

The host protective response against* Leishmania spp.* is predominantly mediated by cellular immunity mechanisms, which are critical for parasite replication control and disease resolution. Initially, during infection, activated dendritic cells (DCs) modulate inflammatory leucocyte recruitment to the infection foci [1] and the development of the T CD4^+^ lymphocyte response characterized by robust IFN-*γ* and IL-17 production [2, 3]. The immune cell recruitment to* Leishmania* infection foci is managed by inflammatory mediators. Chemokines and cytokines have crucial roles in determining the outcome of leishmaniasis [4, 5]. Lipid mediators such as leukotrienes (LTs) are another class of molecules involved in host defense [6].

LTs are generated from the membrane phospholipids of activated innate immune cells, arachidonic acid (AA), through activation of the 5-lipoxygenase (5-LO) enzymes. 5-LO catalyzes oxidation of AA to intermediate 5-hydroperoxyeicosatetraenoic acid (5-HPETE), which is enzymatically reduced by 5-LO to the unstable epoxide A_4_ leukotriene (LTA_4_). LTA_4_ could be hydrolyzed to form leukotriene B_4_ (LTB_4_), which is a potent effector of leukocyte chemotaxis and activation through the BLT_1_/BLT_2_ G-protein coupled receptors [7]. Inflammatory disease such as asthma [8], allergic rhinitis [9], and rheumatoid arthritis [10] are associated with increased levels of LTB_4_.

Studies have demonstrated that LTB_4_ is a potent leukotropic, proinflammatory, and immunoregulator mediator [11, 12]. These molecules are involved in the control of infectious diseases, including viral [13, 14], bacterial [15–17], fungal [18–20], and protozoan infections such as those caused by* T. gondii* [21] and* T. cruzi* [22] and nematode infections [23].

Regarding leishmaniasis, LTB_4_ displays leishmanicidal activity on macrophages [24] and neutrophils [25] during* in vitro* infection with* L. amazonensis*, through mechanisms dependent on nitric oxide (NO) and reactive oxygen species (ROS), respectively. In addition, the inhibition of the 5-LO pathway promoted high susceptibility to* L. amazonensis* infection, increasing footpad swelling and harbored more parasites in resistant and susceptible infected mice [26]. In* in vitro* macrophage infection with* L. donovani *parasites, the 5-LO enzymatic activity is enhanced, leading to increased amounts of arachidonic acid metabolites [27], and* in vivo, L. donovani* infection promotes an increase of cyclooxygenase and lipoxygenase activities in spleen cells [28]. It was recently reported that* L. infantum in vitro *infection inhibits the LTB_4_ signaling pathway dependent on homologous DCSIGN (SIGNR3) during parasite recognition by macrophages [29], suggesting a protective role of LTB_4_ during VL induced by* L. infantum*. Their potential in the recruitment of leukocytes that might be involved in parasite restriction is less well understood. We investigated the role of 5-LO activity in the control of experimental VL induced by* L. infantum, *focusing on the inflammatory immune response. We demonstrated that mice lacking 5-LO signaling displayed high susceptibility to* L. infantum* infection because of a commitment on the related Th17 axis released by CD4 T lymphocytes and neutrophil migration to the infection foci.

## 2. Material and Methods

### 2.1. Mice

Female wild-type 129/SvEv (WT) mice or mice genetically deficient in 5-LO (129/SvEv-5-LO^−/−^), 18–22 g in weight, were housed in the animal facility of the Department of Biochemistry and Immunology of the School of Medicine of Ribeirão Preto at the University of São Paulo (Brazil) in temperature-controlled rooms (22–25°C); the mice received water and food ad libitum. The experiments were conducted in accordance with the National Institutes of Health (NIH) guidelines on the welfare of experimental animals and with the approval of the Ethics Committee of the School of Medicine of Ribeirão Preto.

### 2.2. Parasite Culture, Infection, and Parasite Load Estimation

*L. infantum* (isolate HU-UFS14) was cultured in Schneider medium with 20% heat-inactivated fetal bovine serum, 5% penicillin and streptomycin (from Sigma-Aldrich, Saint Louis, MO, USA), and 2% male human urine. The parasite virulence was maintained by serial passages in BALB/c mice. The mice were injected in the retroorbital plexus with 10^7^ stationary-phase* L. infantum* promastigotes in 100 *μ*L PBS. The hepatic and splenic parasite burdens were determined using a quantitative limiting dilution assay.

### 2.3. DC Generation and Infection

Generation of bone marrow-derived dendritic cells (BMDC) was performed as previously described [30]. The BMDCs (1 × 10^6^/mL) cultured in RPMI-1640 supplemented with 10% FBS were infected with* L. infantum* promastigote forms at a 1 : 5 ratio (cells/parasites) for 12, 24, 36, and 48 h. The supernatants were collected to measure LTB_4_ by ELISA (BiotrakTm, Amersham Pharmacia Biotech, UK). In some wells, LPS (200 ng/mL) was added to the BMDC culture as the positive control group. The cells were harvested and their surface expression characterized by flow cytometry using antibodies against CD11c, MHC class-II, CD86, and CD40 conjugated to APC, FITC, PECy7, PerCP, and Alexa700, respectively, as well as the control isotypes. The cytokine releases were measured into the supernatant culture using commercial ELISA kits, according to the manufacturer's instructions (BD Biosciences, R&D Systems, Minneapolis, MN, USA). In some experiments, selective BLT_1_ leukotriene B_4_ receptor antagonist (U-75302, Sigma-Adrich) (10 *μ*M) was added 12 h before* L. infantum* infection.

### 2.4. Cytokine Release

To assess the influence of LTB_4_ on cytokine production, the liver tissue samples were harvested by a tissue trimmer, weighed, and tittered in 0.5 mL of PBS Complete (Roche Diagnostics, Mannheim, Germany) containing protease inhibitor cocktail. The levels of IFN-*γ*, IL-17, TNF-*α*, IL-12p40, IL-23, IL-6, TGF-*β*, and IL-1*β* were determined using commercial ELISA kits.

### 2.5. Cell Culture and Inflammatory Cells Phenotype

Single-cell suspensions of spleen tissue samples from the 5-LO^−/−^ or WT mice at 6th wpi were aseptically prepared, diluted to a concentration of 2 × 10^6^ cells/mL, and dispensed into 48-well plates in a total volume of 500 *μ*L of complete RPMI-1640 medium (1 × 10^6^ cells/well; Gibco) with or without soluble* Leishmania* Ag (5 *μ*g/mL). The cell culture supernatants were harvested after 72 h of culture at 37°C in 5% CO_2_, and the cytokine levels in the supernatants were determined by ELISA with commercial kits (BD Biosciences and R&D Systems). For the leukocyte identification, the inflammatory cells were gated based on their characteristic size (FSC) and granularity (SSC), and the T lymphocytes (CD4^+^CD3^+^), dendritic cell activation markers (CD11c^high^CD40^+^, CD11c^high^CD86^+^, and CD11c^high^MHC-II^+^), and neutrophils subsets: activated (Ly6G^high^CD11b^high^) or inactivated neutrophils (Ly6G^int⁡^CD11b^int⁡^) were identified individually. For the intracellular staining, the cells were previously cultured with PMA (50 ng/mL) and ionomycin for 4 h in order to obtain the maximum of cytokine production and permeabilized with a Cytofix/Cytoperm kit (BD Biosciences) according to the manufacturer's guidelines and stained with anti-IFN-*γ* or anti-IL-17 conjugated to APC-Cy7 and Alexa700 and with anti-CD3 and anti-CD4 for surface staining with FITC and PerCP, respectively. Rat IgG2b and rat IgG2a were used as the isotype controls. All the antibodies were supplied from BD Biosciences and eBiosciences (San Diego, CA, USA). The cell acquisition was performed using a FACSort flow cytometer. The data were plotted and analyzed using the FlowJo software (Tree Star, Ashland, OR). The total leucocytes counts were determined by relative expression of leucocytes subpopulation stained with specific antibody obtained in 300,000 events acquired and proportional to the leukocytes number obtained in Neubauer chamber.

### 2.6. Statistical Analysis

The data are expressed as the mean ± SEM and are representative of 2–4 independent experiments. The results from the individual experiments were not combined because they were analyzed individually. The means from the groups were compared by ANOVA followed by Tukey's honest significant difference (HSD) test. Statistical significance was set at *P* < 0.05.

## 3. Results

### 3.1. 5-LO Activation Is Required for Experimental* L. infantum* Infection Control

**Figure 1:**
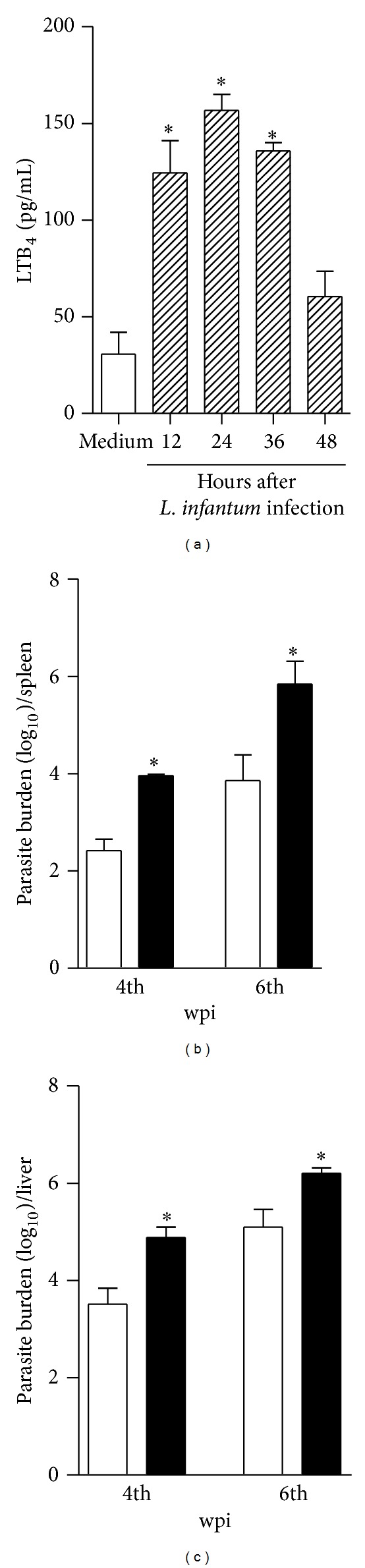
5-LO activity participates in the control of* L. infantum* infection. In (a), the WT BMDCs (1 × 10^6^ cells/mL) were infected with* L. infantum* (1 : 5) (hatched bar) or medium (white bar) for 12, 24, 36, and 48 h and the LTB_4_ amount in the supernatant was determined by ELISA assay. The parasite burden in the spleen (b) and liver (c) was determined in the WT (white bars) and 5-LO^−/−^ (black bars) mice at the 4th and 6th wpi with* L. infantum *promastigote forms (1 × 10^7^ parasites/mice-i.v. route). The data are expressed as the mean ± SEM,* N* = 5-6. *P < 0.05 compared with the WT group.

To determine whether* Leishmania infantum* drives the activation of 5-LO pathway, we performed a kinetic in the release of LTB_4_ by bone marrow-derived dendritic cells after 12, 24, 36, or 48 hours of parasite infection.* L. infantum* induces significant amounts of LTB_4_ by BMDCs at 12 hours postinfection, peaked at 24 hours, and persisted for 36 hours. At 48 hours, the heightened levels of LTB_4_ production were significantly reduced and similar that produced for uninfected cells (medium stimuli) (Figure 1(a)). To characterize the LTB_4_ function, 5-LO^−/−^ and littermate control mice were infected with* L. infantum* and the course of infection was monitored by parasite quantification into the organs by a limiting dilution. We observed that WT presented progressive parasite titers into the spleen (Figure 1(b)) and the liver (Figure 1(c)) over time. The 5-LO^−/−^ mice were more susceptible to infection, harboring more parasites in both target organs than were the WT animals in all the analyzed periods, demonstrating that 5-LO activity, and possibly LTB_4_, participates in the control of* L. infantum.*

### 3.2. *L. infantum*-Infected 5-LO^−/−^ Mice Fail to Recruit Neutrophils to Infectious Foci

Because LTB_4_ presented potent neutrophil chemotactic activity and we and others reported the role of neutrophils in the control of* Leishmania spp.* [11], we characterized the neutrophils present in the spleens of the 5-LO^−/−^ or WT infected mice at 6th wpi.

**Figure 2:**
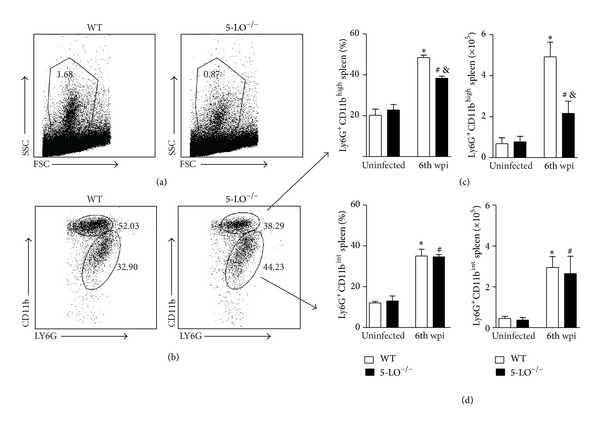
Lack of 5-LO interferes on neutrophil migration. The neutrophils were gated based on their characteristic size (FSC) and granularity (SSC) (a). The dot plots represent the frequency of neutrophils population characterized by LY6G^high^CD11b^high^ (upper gate) and LY6G^int⁡^CD11b^int⁡^ (lower gate) by flow cytometry (b). The bar graphs display the percentage and the absolute number characterized as LY6G^high^CD11b^high^ population (c) or LY6G^int⁡^CD11b^int⁡^ (d) in the spleen from the WT and 5-LO^−/−^ mice at the 6th wpi or from uninfected mice (naïve mice). The data are expressed as the mean ± SEM,* N* = 5-6. *P < 0.05 compared with the WT naïve group, ^&^ *P* < 0.05 compared with the WT infected group, and ^#^ *P* < 0.05 compared with 5-LO^−/−^ naïve group.

Based on their characteristic size (FSC) and granularity (SSC), we observed a significant reduction of cells when analyzed in the granulocytes gate from infected 5-LO^−/−^ mice. Phenotyping the cells, we found that the frequencies of Ly6G^+^CD11b^high^ were present in the spleen samples from the WT infected mice. The percentage of the influx of neutrophils was affected in the 5-LO^−/−^ infected mice, which showed an approximately 30% reduction compared with that of the WT infected mice (Figures 2(b) and 2(c)). In terms of total numbers, the neutrophil reduction was ~50% in the 5-LO^−/−^ mice (Figure 2(c)). We also observed another neutrophils population, LY6G^+^CD11b^interm^ (Figure 2(b)), features of inactive neutrophils since CD11b is upregulated under proinflammatory stimuli [31, 32]. However, their frequency and total cells (Figure 2(d)) in the spleens were similar in both groups. These findings suggest that 5-LO activity participates in neutrophil recruitment to inflammatory foci and, under appropriated activation, might be required for parasite control during* L. infantum* infection.

### 3.3. 5-LO Activity Is Associated with the Development of Host Protective Th17 Responses

**Figure 3:**
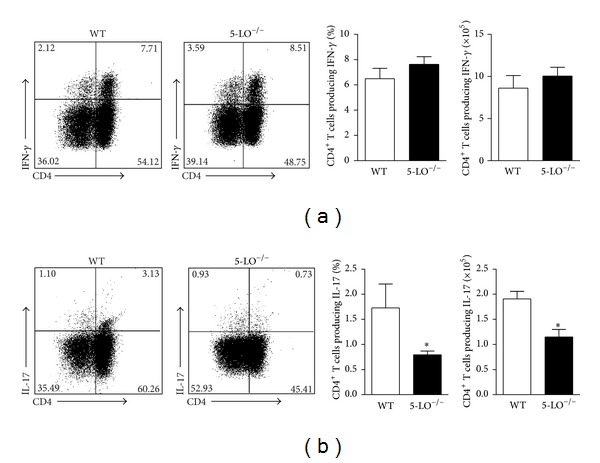
5-LO ablation decreased the Th17, but not the Th1, pattern of immune response. The spleen cells from the WT (white bars) or 5-LO^−/−^ (black bars) mice at the 6th wpi were* in vitro* restimulated with PMA and ionomycin for 4 h and analyzed for intracellular cytokine production by flow cytometry. The dot plots represent the frequency of the CD4^+^ T cell-producing IFN-*γ* (a) and the CD4^+^ T cell-producing IL-17 (b), and the graph bars represent the percentage and the total number of these cells. The data are expressed as the mean ± SEM,* N* = 5-6. *P < 0.05 compared with the WT group.

Because the development of IFN-*γ* and IL-17-producing CD4^+^ T helper cells is crucial for the control of parasite replication in the target organs of LV, we investigated whether these responses were generated in a 5-LO dependent manner. Spleen cells from WT and 5-LO^−/−^ mice at 6th wpi or naïve were* in vitro* restimulated with polyclonal PMA plus ionomycin and the intracellular cytokine production was analyzed. There was no difference in the frequency and absolute number of the IFN-*γ*-producing CD4^+^ T cells in the WT and 5-LO^−/−^ mice (Figure 3(a)). The IL-17-producing CD4^+^ T cells were significantly impaired in the spleens of the 5-LO^−/−^ mice (Figure 3(b)), where the Th17 cells reduction was approximately 50% of that in the WT mice.

**Figure 4:**
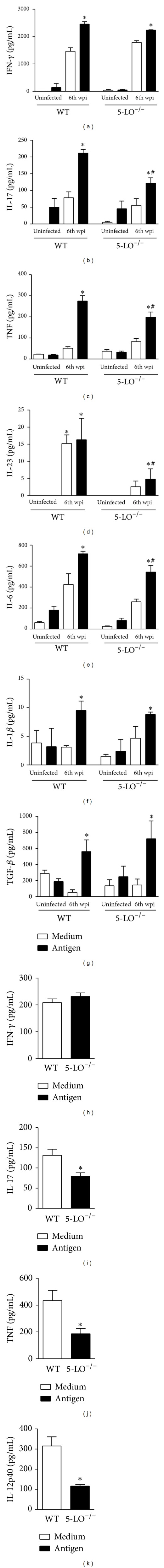
The absence of 5-LO affected the cytokine release related to Th17 pattern. The spleen cells from the WT and 5-LO^−/−^ mice at the 6th week pi or uninfected were* in vitro* stimulated with the* L. infantum* antigen (50 *μ*g/mL) or medium for 72 hours, and the levels of IFN-*γ* (a), IL-17 (b), TNF (c), IL-23 (d), IL-6 (e), IL-1*β* (f), and TGF-*β* (g) were measured in the culture supernatants by ELISA assay. The data are expressed as the mean ± SEM and one representative of two independent experiments. *P < 0.05 compared to the medium; ^#^ *P* < 0.05 compared with the WT stimulation. The liver fragments from the WT (white bar) or 5-LO^−/−^ (black bar) at the 6th wpi with the* L. infantum* promastigote forms were collected and weighed for the determination of IFN-*γ* (h), IL-17 (i), TNF (j), and IL-12p40 (k) by ELISA in the homogenate supernatants. The data are expressed as the mean ± SEM,* N* = 5-6. *P < 0.05 compared with the WT group.

Having determined that 5-LO activity participates in the development of the Th17 response, we measured the production of cytokines in the culture supernatant of the total splenic cells from the WT, 5-LO^−/−^ naïve, or infected mice at 6th wpi and restimulated them* in vitro* with soluble* Leishmania* Ag (SLA). The stimulation with SLA did not induce significant amounts of IFN-*γ* (Figure 4(a)), IL-17 (Figure 4(b)), TNF-α (Figure 4(c)), IL-23 (Figure 4(d)), IL-6 (Figure 4(e)), IL-1*β* (Figure 4(f)), and TGF-*β* (Figure 4(g)) in the culture supernatants of spleens cells from the naïve WT mice compared with those induced in the control (medium). A similar effect was observed in cells from the 5-LO^−/−^ naïve mice when stimulated with the antigen. The infection promoted pronounced levels of all the analyzed cytokines after the SLA stimulation in the WT group, compared to those in the medium (Figures 4(a)–4(g)). Infection in the 5-LO^−/−^ mice resulted in a reduction of cytokine release related to the Th17 axis such as IL-17, TNF-α, IL-23, and IL-6 (Figures 4(b)–4(e)); however, neither IFN-*γ* (Figure 4(a)), IL-1*β* (Figure 4(f)), nor TGF-*β* (Figure 4(g)) productions were affected by a specific stimulus, compared to that obtained in the infected WT mice when stimulated with SLA. Additionally, proinflammatory cytokines in the liver involved in the Th17 axis such as IL-17 (Figure 4(i)), TNF (Figure 4(j)), and IL-12p40 (IL-23) (Figure 4(k)) were reduced in the absence of 5-LO. Corroborating to Figures 3(a) and 4(a), IFN-*γ* amounts were not altered in the deficient mice (Figure 4(h)). These data suggest that 5-LO activity is associated with Th17 response development, and this pathway might be involved in the neutrophils recruitment to inflammatory foci.

### 3.4. DCs Activation May Be Related to 5-LO Activity during Parasite Infection

**Figure 5:**
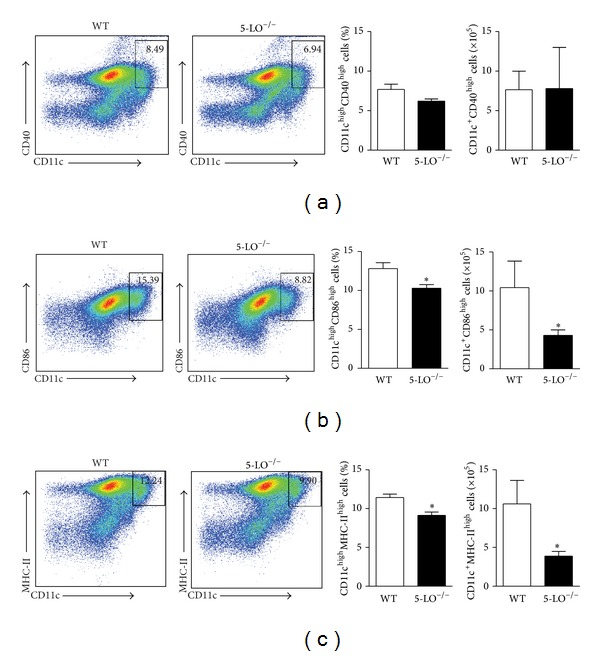
5-LO activity is required for dendritic cell activation into the inflammation site. The* in vivo* surface markers of DCs from the WT (white bars) or 5-LO^−/−^ (black bars) infected mice at the 6th wpi were determined by flow cytometry. The dot plots represent the frequency of CD40 (a), CD86 (b), and MHC-II (c) in the CD11c^high^ population. All analyses were performed on CD11b^+^CD11c^high^ gated cells. The data are expressed as the mean ± SEM,* N* = 5-6. *P < 0.05 compared to the WT group.

**Figure 6:**
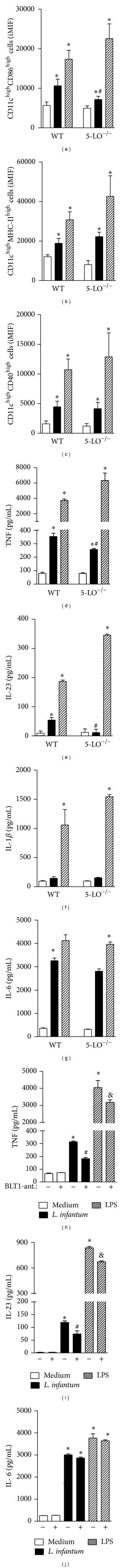
The absence of 5-LO interferes with BMDC maturation and the release of innate cytokines induced by* L. infantum* through BLT_1_ receptor. The WT or 5-LO^−/−^ BMDC was stimulated with* L. infantum* (5 : 1) (black bars), LPS (200 ng/mL) (hatched bars), or medium (white bars) for 24 h. The BMDCs were harvested and the costimulatory molecules expression such CD86 (a), MHC-II (b), and CD40 (c) was evaluated by flow cytometry. All analyses were performed for the CD11c^high^ population. The TNF (d), IL-23 (e), IL-1*β* (f), and IL-6 (g) levels were measured in the supernatant of the BMDC culture by ELISA assay. The data are expressed as the mean ± SEM and are representative of three independent experiments. *P < 0.05 compared with the medium; ^#^ *P* < 0.05 compared with the infected WT. ^&^ *P* < 0.05 compared with the infected WT. In some experiment, selective BLT_1_ leukotriene B_4_ receptor antagonist (10 *μ*M) was or not added 12 h before* L. infantum* infection or LPS stimuli. The levels of TNF (h), IL-23 (i), and IL-6 (j) were determinate into supernatant 24 thereafter. *P < 0.05 compared with the medium; ^#^ *P* < 0.05 compared with* L. infantum* infection. ^&^ *P* < 0.05 compared with LPS stimuli.

Because dendritic cells (DCs) are the main cells involved in orchestrating immune responses during* Leishmania sp.* infection through the release of cytokines which might be involved in the differentiation of Th17 cells [33], we first evaluated, using flow cytometry analyses, the maturation profile of dendritic splenic cells of the WT and 5-LO^−/−^ mice infected at 6th wpi by evaluating the costimulatory molecules in the CD11c^high^ cells. In terms of percentage, the DC expressing CD86 (Figure 5(b)) or MHC-II (Figure 5(c)) is slightly reduced in the absence of 5-LO that was ~20% less compared with WT. However, in terms of total cells, we observed a markedly reduction of DCs expressing CD86 or MHC-II that was ~50% into spleens of 5-LO^−/−^ mice (Figures 5(b)-5(c), resp.). No difference was observed in the CD40 expression (Figure 5(a)). Consistent with the* in vivo* data, the bone marrow-derived DC (BMDC) from the WT infected* in vitro* with parasites enhanced the expression levels of surface markers such as MHC-II, CD40, and CD86 (Figures 6(a)–6(c)), when compared to those of the medium. In contrast, infection of BMDC from 5-LO^−/−^ with* L. infantum* inhibits their activation, presenting reduced expression of CD86 surface markers (Figure 6(c)). The absence of 5-LO did not alter the LPS-induced dendritic cell maturation (Figures 6(a)–6(c)).

Next, we evaluated the release of innate cytokines involved in Th17 axis differentiation by DCs. Thus, we determined the levels of TNF, IL-23, IL-1*β*, and IL-6 in the supernatants from the WT or 5-LO^−/−^ BMDCs cultured with* L. infantum* parasites or medium. As the positive control, the cells were activated with LPS. The parasites induced significant production of TNF (Figure 6(d)), IL-23 (Figure 6(e)), IL-1*β* (Figure 6(f)), and IL-6 (Figure 6(g)) by the DC from WT when compared with that of the respective control group. Additionally, the parasites promoted significant amounts of cytokines in the DC from 5-LO^−/−^, compared to the 5-LO^−/−^ DC stimulated with the medium; however TNF (Figure 6(d)), IL-23 (Figure 6(e)), and IL-6 (Figure 6(g)) levels were significantly decreased comparing those released by infected WT DC. The levels of IL-1*β* (Figure 6(f)) were unaltered in the absence of 5-LO. These data suggest that 5-LO participates in DC activation, interfering with the cytokine release involved in the Th17 subset polarization during an experimental* L. infantum* infection.

The ablation of 5-LO lacks not only LTB_4_, but also cysteinyl leukotrienes including LTC_4_, LTD_4_, and LTE_4_ activity [34]. In other to clarify, in part, the effect of LTB_4_ during* L. infantum* infection, we use* in vitro* a selective BLT_1_ leukotriene B_4_ receptor antagonist (U-75302). The BLT_1_ antagonist was added to BMDC culture 12 h before* L. infantum* infection and the release of cytokines related to Th17 pattern was measured into culture supernatant by ELISA assay. As expected, TNF (Figure 6(h)), IL-23 (Figure 6(i)), and IL-6 (Figure 6(j)) were produced during infection. The ability of BMDCs infected with parasites to produce cytokines such as TNF (Figure 6(h)) and IL-23 (Figure 6(i)), but not IL-6 (Figure 6(j)), was inhibited by BLT_1_ blockage, confirming that LTB_4_ is associated with the release of cytokines involved in the Th17 axis. We do not rule out the possibility of others leukotrienes that may contribute to cytokine release, herein, that is, IL-6 release, but we undoubtedly evidenced LTB_4_ participation in the control of VL.

## 4. Discussion

In this study, we report 5-LO activity, and presumably LTB_4_, as an important mediator in controlling infection induced by* Leishmania infantum*. This eicosanoid that is released during infection may promote the activation of dendritic cells, which influence the release of mediators involved in the drive of naive CD4^+^ T lymphocytes to the Th17 profile. In the last instance, the Th17 subtype recruits neutrophils to the infection foci that might retrain the parasite restriction. Understanding the role of LTB_4_ in the inflammatory process mediated by* L. infantum* might elucidate some of the effector mechanisms that control the replication of the parasites.

We demonstrated that infection with* L. infantum* results in the production of LTB_4_ by dendritic cells during* in vitro* infection. The absence of endogenous LTB_4_ promoted higher susceptibility to infection. The genetic ablation of 5-LO harbored more parasites in target organs such as the spleen and liver, demonstrating its role in the control of infection. These results are consistent with those of previous studies that demonstrate the role of LTB_4_ in the control of infectious processes [13, 17, 21], increasing the leishmanicidal activity of macrophages [24] and of neutrophils [25] by a nitric oxide (NO)-dependent mechanism and release of reactive oxygen species (ROS), respectively.

Several studies have demonstrated that LTB_4_ is a potent inducer of neutrophils. During leishmaniasis, neutrophils are rapidly mobilized to the inflammatory site [1, 35], where they eliminate the pathogen by the production of reactive oxygen species (ROS) and the release of peptides and antimicrobial proteases [36–38]. In our results, the high susceptibility observed in animal 5-LO^−/−^ was accompanied by the failure of neutrophil migration. LTB_4_ has a central role in controlling the migration of neutrophils to sites of inflammation through BLT_1_ and BLT_2_ (leukotriene receptors) [39], directly by inducing the expression of the CD11b and CD18 integrins [40] or indirectly by amplifying the production of inflammatory mediators such as cytokines and chemokines by others cells [41, 42]. In fact, we observed a significant reduction of activated neutrophils expressing CD11b into target organs that were infected by parasites in the absence of 5-LO. Furthermore, LTB_4_ enhances effectors mechanisms of neutrophils such as phagocytic capacity [43] and granules releasing and stimulates the enzymatic generation of ROS [14, 44, 45], including* in vitro* infection by* L. amazonensis *[25]. Thus, it seems that the protector role of LTB_4_ during LV may be played by the recruitment and activation of neutrophils to the site of infection.

The recruitment of neutrophils might be induced by cytokines such as IL-17 because they are potent granulopoietic factors [46] that induce the release of CXC chemokines [47]. We found that the absence of LTB_4_ synthesis impaired the Th17 response, whereas the Th1 response was unchanged in the target organs. Consistently, the production of IL-17 by spleen cells in response to the specific stimulus (i.e.,* Leishmania* antigen) and its detection in the liver of 5-LO^−/−^ infected mice was inhibited, confirming the interference of LTB_4_ in the release of IL-17. We have not evaluated whether LTB_4_ participates in the control of* Leishmania infantum *through Th17-dependent manner; however, we believe that the administration of recombinant IL-17 may rescue the protective effect of leucotrienes in susceptible 5-LO deficient mice. In fact, administration of recombinant IL-17 or IL-23 in susceptible BALB/c mice infected with* L. donovani *controlled parasite replication, which was associated with increased iNOS activity [3]. Furthermore, exogenous LTB_4_ is able to positively modulate the differentiation of Th17 cells from naive CD4^+^ T cells [48]. The induction of experimental autoimmune encephalomyelitis (EAE) in animals genetically deficient in BLT_1_ presented clinical score signs attenuated because of impairment of the Th17 generated response. Infiltration of T cells, macrophages, and granulocytes into the spinal cord was reduced in the BLT_1_^−/−^ mice [49], demonstrating the involvement of LTB_4_ in the development of the Th17 response.

LTB_4_ is produced during inflammatory and infectious processes by several leucocytes [50], including activated neutrophils, macrophages, and T cells [51–54]. Among the cells able to synthesize LTB_4_, DCs play an important role in the initiation of immune responses because they are the main cells involved in pathogen recognition, triggering several proinflammatory mechanisms that bridge to adaptative immune responses [55–57]. According to our results, DCs are potential sources of LTB_4_ during* L. infantum* infection. Given the importance of the role lipid mediators play in leucocyte activation, LTB_4_ production by DCs is a major mechanism for the modulation of the effector function of other cell subsets during LV, for example, mediating the recruitment of neutrophils to inflammation sites. We do not rule out the possibility of others leukotrienes that may contribute to cytokine release, since that the ablation of 5-LO lacks not only LTB_4_ but also cysteinyl leukotrienes including LTC_4_, LTD_4_, and LTE_4_ activity [34]. However, the pharmacological blockage of BLT_1_ prevented, at least in part, the release of cytokines by DC, evidencing LTB_4_ association with Th17 axis, and in last instance, controlling parasite replication.

Apart from sources of LTB_4_, DCs are the target of the action of lipid mediators as an important mechanism for modulating the immune response [58, 59]. An impaired Th17 response might result from failed DC activation in the absence of 5-LO. This hypothesis might be supported by the following explanations. First, exploring the role of LTB_4_ in DC activation, our data demonstrated that the maturation phenotype of DCs from animal 5-LO^−/−^ was reduced during* in vivo* and* in vitro* infection. Consistently, the addition of LTB_4_ in cultured BMDCs induces maturation of these cells to increase MHC-II expression. Blockage of 5-LO with NDGA protects cells from the effects of LTB_4_ on DC maturation [60]. BMDCs migrate and are activated in response to LTB_4,_ and its effect is lost in cells that lack BLT_1_ [61]. LTB_4_ upregulates the expression of CCR7 and its ligand CCL19/ELC, which mediate the migration to lymphoid organs. Second, the impaired ability of DCs from animal 5-LO^−/−^ to secrete cytokines is involved in the polarization of naïve CD4 T cells to the Th17 profile. Naïve CD4^+^ T lymphocytes are polarized to the Th17 subset through the combined pattern of the action of cytokines such as IL-1*β*, TGF-*β* and IL-6 [62], whereas activation requires sustained stimulation with IL-23, which is predominantly produced by dendritic cells and TNF release [63]. Our data demonstrated that the production of TNF, IL-23, and IL-6* in vivo,* at least, was compromised in the absence of 5-LO. Supporting our hypothesis, a significant reduction of IL-23, TNF, and IL-6 by BMDC was observed in the dendritic cells derived from animal 5-LO^−/−^. Consistently, Lefèvre and colleagues demonstrated that cytokines such as IL-1*β*, TGF-*β*, and IL-6 are highly produced by macrophages infected* in vitro* with* L. infantum *[29]. The role of LTB_4_ in the induction of innate cytokines related to the Th17 profile differentiation released by DCs is unprecedented. It is known that IL-1R signaling is dependent on the BLT_1_ downstream pathway. The requirement for the BLT_1_ signaling pathway is overcome by exogenous administration of IL-1*β* in LTB_4_^−/−^ mice [64]. Moreover, BLT_1_ expression is upregulated in Th17-differentiated T cells [49] and* ex vivo* studies have demonstrated that the production of TNF and IL-6 was impaired in the absence of BLT_1_^−/−^ cells [65, 66], confirming the role of LTB_4_ in driving the Th17 response.

We do not investigate the molecular mechanisms by which 5-LO activity interferes with maturation process and subsequent activation of dendritic cells, but we believe that the initial response is dependent on TLR4 signaling. During parasite recognition through TLR4 pathway, the adapter molecule MyD88 is recruited and activates factors such as NF-*κ*B [67, 68], leading to transcription of proinflammatory cytokines such as TNF, IL-6, and IL-23. MYD88 recruitment also activated 5-LO enzyme, promoting the synthesis of leucotriens, especially LTB_4_ that, through BLT_1_ pathway, amplifies the activation of NF-*κ*B which may induce cellular activation [66]. Interestingly, genetic deletion of 5-LO or pharmacological blockade of BLT_1_ receptor interferes with the secretion of proinflammatory cytokines by DCs and their maturation phenotype. The 5-LO pathway may act in autocrine manner, increasing the activation and function of DCs, and greatly influence the magnitude response of Th17 cells as well. Thus, the amplification of the inflammatory response mediated by 5-LO activation during parasite recognition by DCs appears to play an important role in controlling parasite replication.

## 5. Conclusion

Our data demonstrated that 5-LO activity, and perhaps LTB_4_, plays a prominent role in controlling* L. infantum*-induced visceral leishmaniasis, which may be associated with the development of the Th17 response and the subsequent recruitment of neutrophils to the inflammatory site that is dependent on dendritic cell activation. Future studies might characterize which innate receptors on DCs are involved in the recognition of the parasite, leading to a subsequent synthesis of LTB_4_. The results show, for the first time, the role of LTB_4_ in the development of the Th17 response in the context of an infectious disease.
